# Phosphorylation of the neurogenic transcription factor SOX11 on serine 133 modulates neuronal morphogenesis

**DOI:** 10.1038/s41598-018-34480-x

**Published:** 2018-11-01

**Authors:** Elli-Anna Balta, Iris Schäffner, Marie-Theres Wittmann, Elisabeth Sock, Felix von Zweydorf, Julia von Wittgenstein, Kathrin Steib, Birgit Heim, Elisabeth Kremmer, Benjamin Martin Häberle, Marius Ueffing, Dieter Chichung Lie, Christian Johannes Gloeckner

**Affiliations:** 1 Institute of Biochemistry, Friedrich-Alexander Universität Erlangen-Nürnberg, 91054 Erlangen, Germany; 2 DZNE-German Center for Neurodegenerative Diseases, 72076 Tübingen, Germany; 3 Institute of Developmental Genetics, Helmholtz Zentrum München, German Research Center for Environmental Health, 85764 Neuherberg, Germany; 4 University of Tübingen, Institute for Ophthalmic Research, Center for Ophthalmology, 72076 Tübingen, Germany; 5 Monoclonal Antibody Core Facility, Helmholtz Zentrum München, German Research Center for Environmental Health, 85764 Neuherberg, Germany

## Abstract

The intellectual disability gene, Sox11, encodes for a critical neurodevelopmental transcription factor with functions in precursor survival, neuronal fate determination, migration and morphogenesis. The mechanisms regulating SOX11’s activity remain largely unknown. Mass spectrometric analysis uncovered that SOX11 can be post-translationally modified by phosphorylation. Here, we report that phosphorylatable serines surrounding the high-mobility group box modulate SOX11’s transcriptional activity. Through Mass Spectrometry (MS), co-immunoprecipitation assays and *in vitro* phosphorylation assays followed by MS we verified that protein kinase A (PKA) interacts with SOX11 and phosphorylates it on S133. *In vivo* replacement of SoxC factors in developing adult-generated hippocampal neurons with SOX11 S133 phospho-mutants indicated that phosphorylation on S133 modulates dendrite development of adult-born dentate granule neurons, while reporter assays suggested that S133 phosphorylation fine-tunes the activation of select target genes. These data provide novel insight into the control of the critical neurodevelopmental regulator SOX11 and imply SOX11 as a mediator of PKA-regulated neuronal development.

## Introduction

The SOXC protein SOX11 is a potent transcriptional regulator, which has been functionally linked to early and late steps of mammalian neurogenesis including neural precursor survival, proliferation, neuronal fate commitment, migration and dendrite development^1–6^. The critical role of SOX11 for human CNS development was predicted by single-cell transcriptomic analysis of human neocortical development^7^ and was confirmed by the discovery that heterozygote mutations in Sox11 are associated with Coffin-Siris Syndrome, a rare human congenital disorder characterized by intellectual disability, microcephaly and growth deficiency^8,9^.

The regulation of SOX11 remains poorly understood. Recent data suggests that SOX11 activity may be controlled not only by epigenetic and transcriptional mechanisms, but also by post-translational modifications. In retinal ganglion cells, SOX11’s subcellular localization is modulated by SUMOylation^10^. In previous work we identified ten candidate serine residues for phosphorylation via mass spectrometry. Notably, we demonstrated that phosphorylation of SOX11 on serine 30 (S30) resulted in the redistribution of SOX11 from an exclusive nuclear localization to a mixed nuclear and cytoplasmic localization^11^.

Here, we focused on the impact of phosphorylation on SOX11’s transcriptional activity and on the identification of kinases controlling SOX11’s function. We show that the three phosphorylatable serine residues surrounding the DNA binding High-mobility group (HMG)-box, i.e., S30, S133, and S137, modulate SOX11’s transcriptional activity. Moreover, we provide evidence that Protein Kinase A (PKA) interacts with SOX11 and phosphorylates SOX11 on S133. Finally, we provide evidence that phosphorylation of SOX11 on S133 modulates dendritic morphogenesis *in vivo*.

## Results

### The first 3 Phospho-serines of SOX11 are important for its transcriptional activity

**Figure 1:**
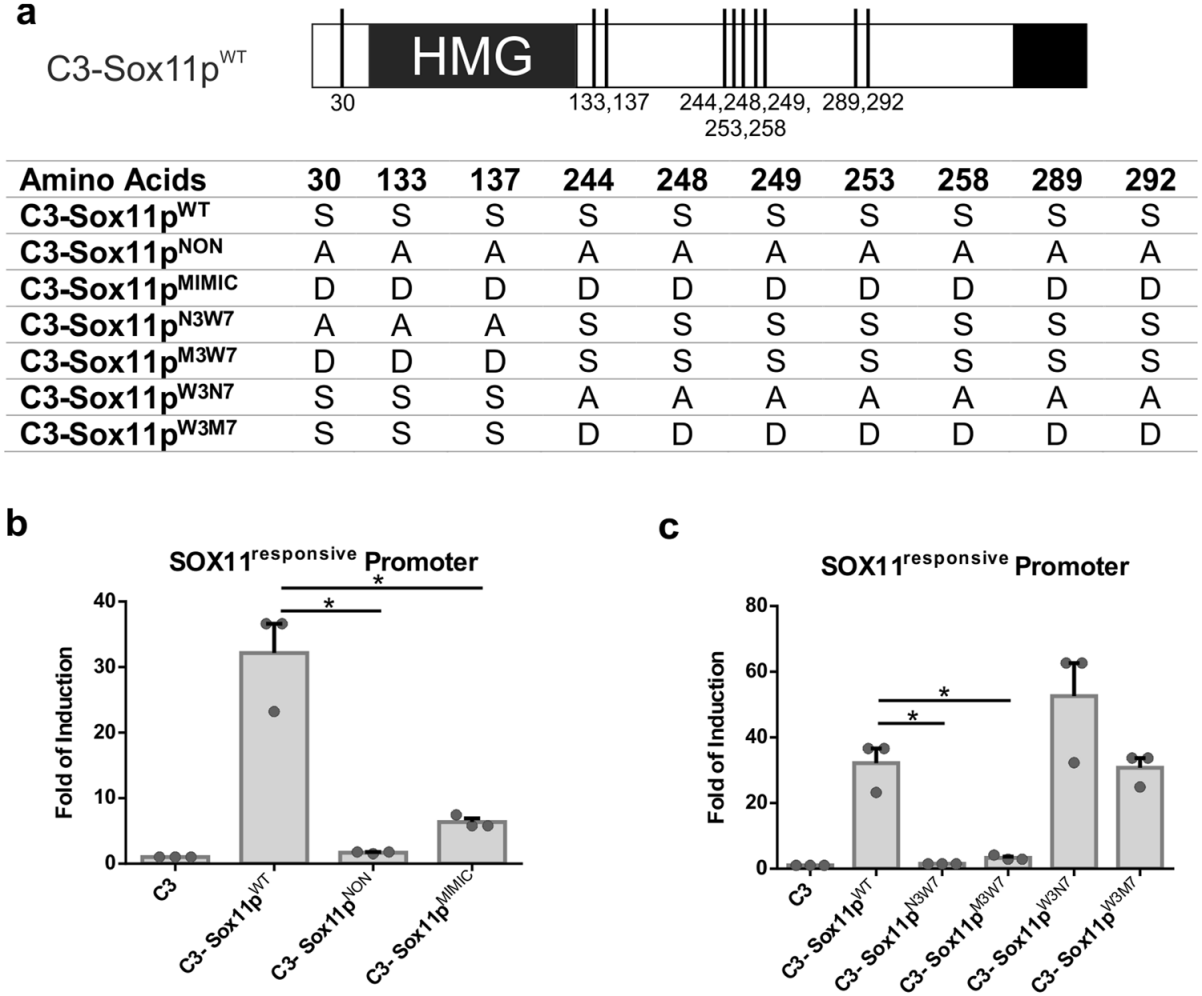
The three phospho-serines surrounding the HMG-box are important for SOX11’s transcriptional activity. (**a**) Representation of the phosphorylated serine residues on SOX11 WT and Table presenting the amino acids that are exchanged on each mutant. S = Serine (wild type), A = Alanine (NON-phosphorylatable aa) and D = Aspartate (aa that mimics phosphorylation). (**b**) Luciferase assays using a SOX11-responsive minimal promoter n = 3, performed in HEK293T cells (Student’s unpaired T-test: WT vs NON p = 0.021, WT vs MIMIC p = 0.027). (**c**) Luciferase assays using a SOX11-responsive minimal promoter n = 3, performed in HEK293T cells (Student’s unpaired T-test: WT vs N3W7 p = 0.021, WT vs M3W7 p = 0.022, WT vs W3N7 p = 0.17, WT vs W3M7 p = 0.81). The data are presented as mean ± SEM.

Our previous mass spectrometry (MS)-based analysis identified ten putatively phosphorylated serine residues in the murine SOX11 protein^11^. Three of these residues, i.e., S30, S133, and S137, surround the DNA-binding HMG-box (Fig. 1a). These serine residues are highly conserved between species and found exclusively in SOX11 but not in other members of the SOXC transcription factor family. The other seven putatively phosphorylated serine residues are located more towards the C-terminus and do not show conservation between species (Fig. 1a)^11^. To investigate the impact of SOX11’s phosphorylations on its transcriptional activity, we tested the ability of SOX11 phosphorylation mutants for transcriptional activation in luciferase assays. Mutations of all phosphorylatable serines to an amino acid (i.e., alanine) that mimics non-phosphorylated serine (C3-Sox11p^NON^) or to an amino acid (i.e., aspartate) that mimics phosphorylation (C3- Sox11p^MIMIC^) abolished transcriptional activation of the SOX11-responsive minimal promoter^12^, which contains multiple binding sites for SOX11 (Fig. 1b).

To clarify, which of the phosphorylatable serines impact on SOX11’s transcriptional activity mutations were separated into two groups. The first group contains the three N-terminal serines flanking the HMG-box while the second group comprised the seven C-terminal serines S244, S248, S249, S253, S258, S289 and S292. In both groups, the serines were mutated to either a non-phosphorylatable or a phospho-mimetic form to generate the following mutants: (i) C3-Sox11p^N3W7^ = NON 3 Wildtype (WT) 7, where the three N-terminal serines are mutated into a non-phosphorylatable form, (ii) C3-Sox11p^M3W7^ = MIMIC 3 WT 7, where the three N-terminal serines are mutated into a phosphomimetic form, (iii) C3-Sox11p^W3N7^ = WT 3 NON 7, where the seven C-terminal serines are mutated into a non-phosphorylatable form, and iv) C3-Sox11p^W3M7^ = WT 3 MIMIC 7, where the seven C-terminal serines are mutated into a phosphomimetic form (Fig. 1a). While transcriptional activation of the SOX11-responsive minimal promoter by the C3-Sox11p^W3N7^ and C3-Sox11p^W3M7^ mutants was comparable to the activation by non-mutated Sox11, neither C3-Sox11p^N3W7^ nor C3-Sox11p^M3W7^ significantly activated the SOX11-responsive minimal promoter (Fig. 1c). These data strongly suggest that the conserved, three N-terminal phosphorylatable serines around the HMG-box, are important for SOX11’s transcriptional activity.

### PKA interacts with SOX11 and phosphorylates its serine 133

**Figure 2:**
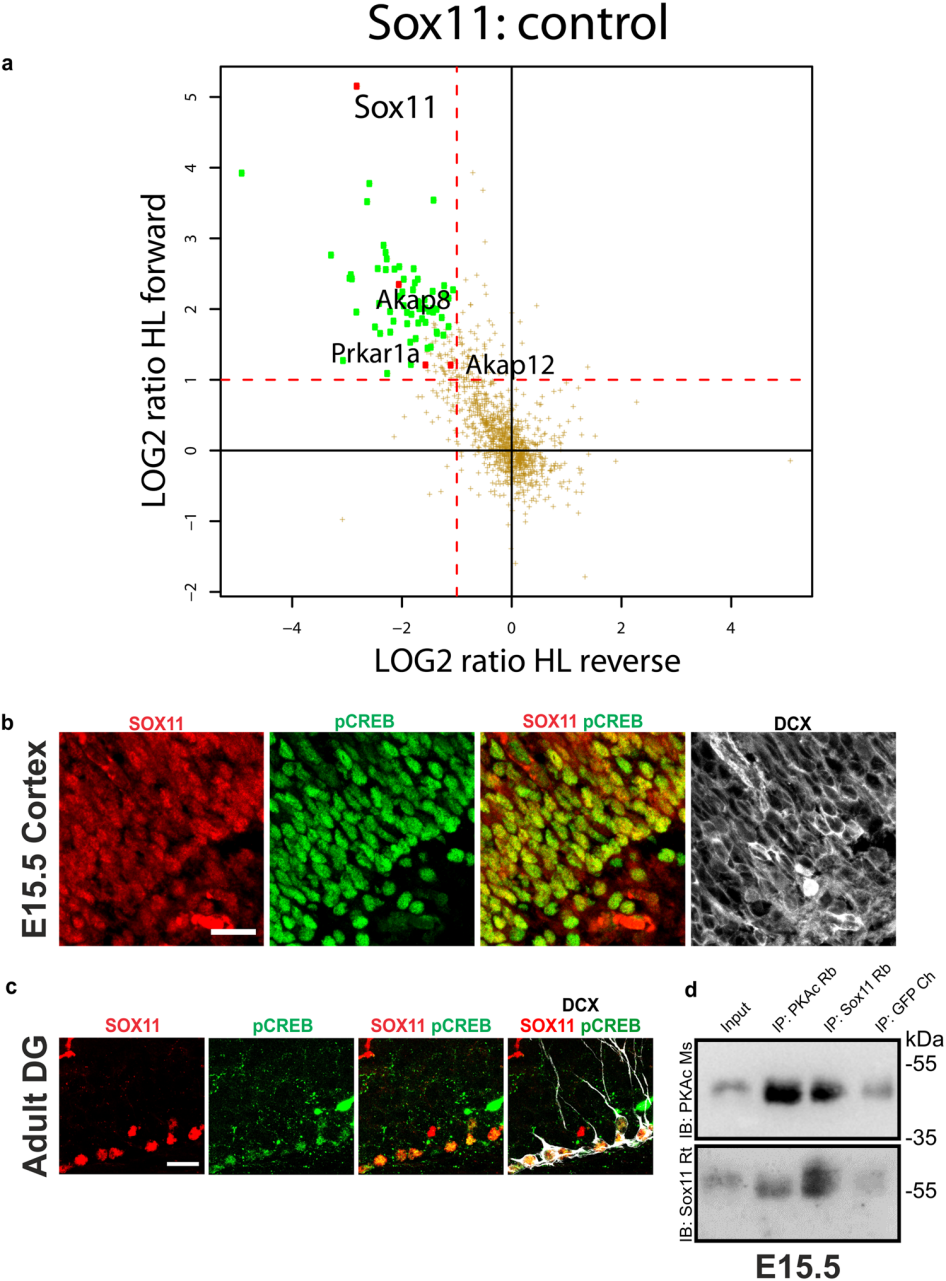
PKA interacts with SOX11. (**a**) Scatter plot showing the outcome of the quantitative MS-based interactome study for Strep/FLAG SOX11 after recombinant expression in Neuro2a cells. The x axis represents the ratios (bait vs control) of the reverse (label switch) SILAC experiments while the ratios of the forward experiments are presented by the y axis. Significant hits with a ratio >2.0 for the forward and <0.5 for the reverse experiments are shown in green. The SOX11 interactors Prkar1a (RIα), AKAP8 and AKAP12, involved in PKA regulation, are highlighted. (**b**) Immunofluorescent analysis of the co-localization of SOX11 and pCREB in the cortex of embryos on E15.5. SOX11 (in red), pCREB (in green), DCX (in grey). Scale bars 50 μm. (**c**) Immunofluorescent analysis of the co-localization of SOX11 and pCREB in the subgranular zone of the dentate gyrus (DG) of adult mice. SOX11 (in red), pCREB (in green), DCX (in grey). Scale bars 20 μm. (**d**) Co-immunoprecipitation assay conducted with brain extracts from embryos on E15.5. Blotting with an anti-PKAc Mouse antibody showed a more intense band in the IP: PKAc sample compared to the Input while the PKAc band in the IP: SOX11 was more intense than the one in the IP: GFP. Blotting with an anti-SOX11 Rat antibody showed that the SOX11 band on the IP: SOX11 was more intense than the one on the Input. Likewise, in the IP: PKAc sample the SOX11 band was more intense when compared with the negative control, IP: GFP. The blots presented are cropped.

To identify kinases mediating SOX11’s phosphorylation, its interactome was determined by AP-MS (affinity purification combined with MS). For this purpose, Strep/FLAG SOX11 was recombinantly expressed in Neuro2A and immunoprecipitated via anti-FLAG antibodies. Co-purified proteins were analyzed by SILAC-based quantitative mass spectrometry to identify specific SOX11 interactors. The full list of interactors is shown in Supplemental Table 1. Interestingly, this analysis identified Protein Kinase A Type 1a Regulatory Subunit (PRKAR1a, RIα) as well as the A-Kinase Anchoring Proteins 8 (AKAP8) and 12 (AKAP12) as interacting partners of SOX11 (Fig. 2a). PKA is a holoenzyme that consists of four subunits, two catalytic (PKA_C_) and two regulatory (PKA_R_). It was identified that different combinations of catalytic and regulatory subunits have distinct biochemical properties being differentially responsive to cAMP^13^. Additionally, PKA’s specificity and versality is regulated by its scaffold proteins, AKAPs. AKAPs form multiprotein complexes that in addition to PKA contain for example phosphatases, which allow immediate signal termination. PKA is also confined to different cellular compartments by AKAPs, thus, localizing PKA in close proximity to its targets^14^. AKAP8 is responsible for cAMP-responsive nuclear events and it has been associated with microcephaly and autism-like phenotypes in humans^15^. Thus, the finding that SOX11 biochemically interacts with PRKAR1a and AKAPs suggests that SOX11 may be targeted by the Protein Kinase A (PKA) signaling pathway.

We next investigated whether PKA is active in SOX11 expressing cells of the developing and adult brain. The transcription factor cyclic AMP response element binding protein (CREB) is a bona fide target of PKA and its phosphorylation on serine 133 is commonly used as a proxy for PKA pathway activity^16^. Immunofluorescent staining of the developing mouse cortex on embryonic day 15.5 (E15.5) and of the adult hippocampal dentate gyrus using antibodies against S133 phosphorylated CREB (pCREB), SOX11, and the immature neuronal marker doublecortin (DCX) showed that SOX11-positive immature neurons were immunoreactive for pCREB. These findings indicate that the PKA pathway is active in SOX11-expressing cells of the embryonic and adult brain (Fig. 2b and c). We also found that SOX11 co-immunoprecipitated with PKAc and vice-versa in E15.5 mouse brain extracts demonstrating that SOX11 and PKA interact *in vivo* (Fig. 2d and Supplemental Fig. 1).

**Figure 3:**
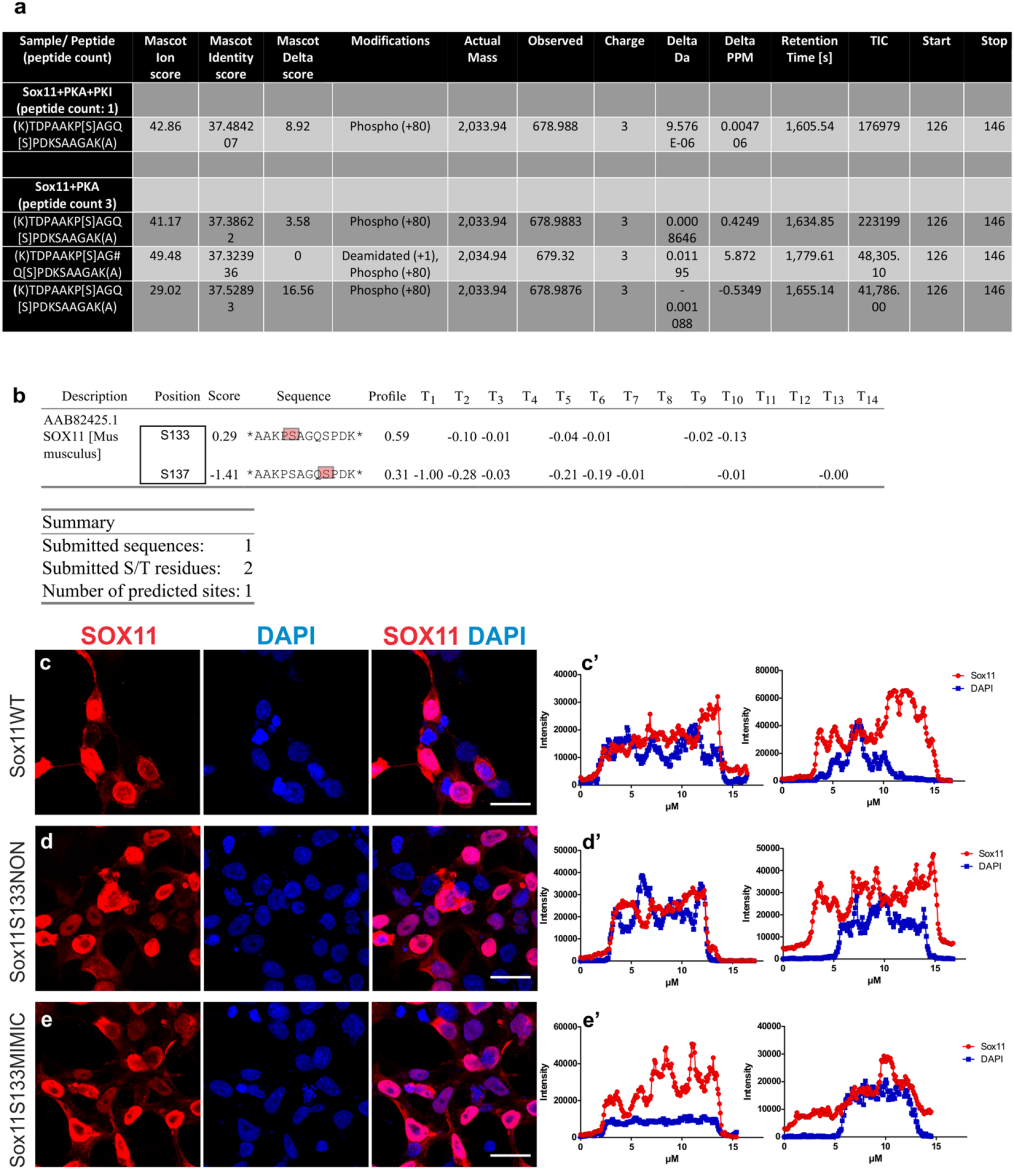
PKA phosphorylates SOX11 in serine 133. (**a**) Mass Spectrometry analysis of the *in vitro* phosphorylation assay. The table reports the spectral data for the phosphopeptide corresponding to Sox11 pS133/137, including the number of spectra with a peptide probability > 50% (Scaffold); the Mascot ion, identity and delta scores; the type of residue modifications, the theoretical (actual) as well as the observed mass; the peptide charge; the delta mass in Dalton and PPM; the retention time, the total ion count (TIC), the start and stop positions within the murine SOX11 amino acid sequence. (**b**) Comparison of the sequence around S133 and S137 with pkaPS. The table reports that PKA is predicted to phosphorylate S133 with score 0.29 but not S137 (score -1.41). Immunofluorescent analysis and line intensity plots of the subcellular localization (**c–**c’) of SOX11WT in HEK293T cells overexpressing pCAG–Sox11WT–IRES–GFP, (**d**–d’) of SOX11S133NON in HEK293T cells overexpressing pCAG–Sox11S133NON–IRES–GFP, and (**e**-e’) of SOX11S133MIMIC in HEK293T cells overexpressing pCAG–Sox11S133MIMIC–IRES–GFP. SOX11 (in red) and DAPI (in blue). Scale bars = 20 μm. (c’–e’) Representative line intensity plots of HEK293T cells transfected with SOX11 wildtype and mutants. The blue line represents the intensity of DAPI, the red line represents the intensity of SOX11 along a cross-section of a cell. The left intensity plot shows a cell with nuclear SOX11 localization (note the overlap between the DAPI and the SOX11 signal), while the right intensity plot shows a cell with nuclear and cytoplasmic SOX11 localization (note that the SOX11 signal extends beyond the DAPI signal).

To identify the serine residue that is phosphorylated by PKA we performed *in vitro* kinase assays of SOX11 followed by MS analysis. Overexpressed SOX11 was immunoprecipitated from HEK293T cells. Precipitated SOX11 was incubated with purified PKAc in the presence or absence of a Protein Kinase A inhibitor peptide (PKI). MS analysis and quantitative assessment by spectral counting revealed increased phosphorylation on a peptide covering the S133 and S137 residue in the presence of PKAc compared to samples additionally treated with PKI (Fig. 3a). Because of the close proximity of the S133 and S137 residues, mass spectrometry could not distinguish which of the serines was phosphorylated. Comparison of the amino-acid sequences surrounding S133 and S137 using a bioinformatical algorithm specifically designed to predict PKA phosphorylation sites (pkaPS)^17^, however, identified S133 as the more probable site for PKA-mediated phosphorylation (Fig. 3b). To test whether S133 influences SOX11’s subcellular localization^11^, we overexpressed Sox11WT, Sox11S133NON (S133ASox11, non-phosphorylatable), and Sox11S133MIMIC (S133DSox11, phosphomimetic), in HEK293T cells and performed immunofluorescent stainings. In both mutants and SOX11WT, immunofluorescent stainings and fluorescent line intensity plots identified cells with nuclear or nuclear and cytoplasmic SOX11 localization (Fig. 3c-e’) suggesting that the phospho-status of SOX11S133 does not influence SOX11’s subcellular localization.

### SOX11S133 mutants differentially modulate dendrite morphology of adult-born dentate granule neurons

**Figure 4:**
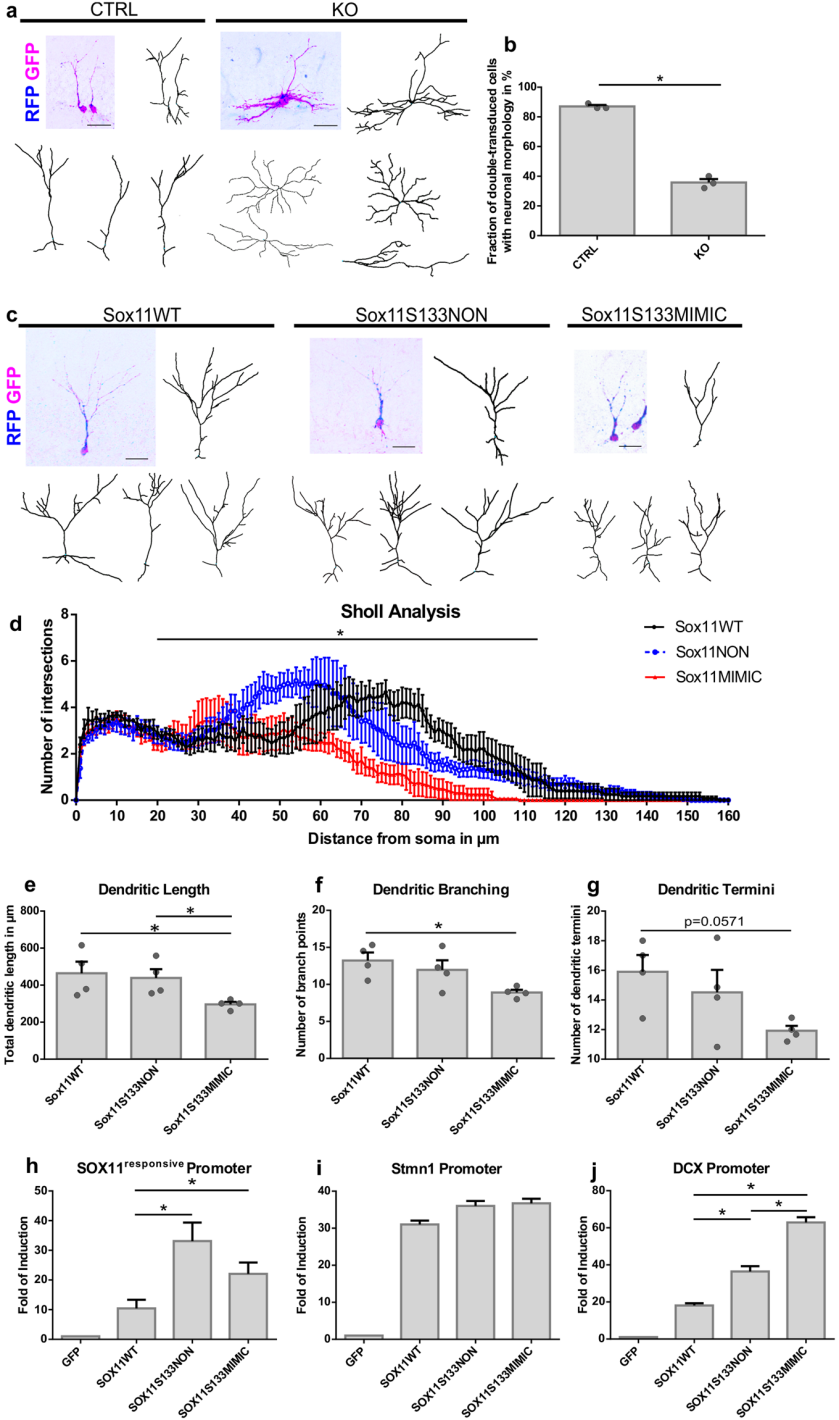
S133 SOX11 mutants differentially modulate the dendritic morphology of adult-born dentate granule neurons. (**a**,**b**) SOXC protein function is required to establish a polarized neuronal morphology. For conditional ablation of Sox4 and Sox11 from neural precursor cells (NPCs) in the dentate gyrus of adult mice, Sox4^loxP/loxP^/Sox11^loxP/loxP^ double conditional knockout mice (Sox4/11 cKO mice) were injected with a 1:1 mixture of the CAG-GFP-IRES-CRE + CAG-mitoDsRed MML-retroviruses (KO). Sox4/11 cKO mice injected with a mixture of the CAG-GFP + CAG-mitoDsRed MML-retroviruses served as control (CTRL). The morphology of the progeny of double-transduced NPCs was analyzed 12 days post injection (dpi). (**a**) Sample reconstructions of cells generated from Sox4/Sox11-deficient NPCs (KO) and control NPCs (CTRL). (**b**) Quantification of cells with neuronal morphology among the cells generated from Sox4/Sox11-deficient NPCs (KO) and control NPCs (CTRL) (n = 3, Student’s unpaired t-test: p < 0.0005). (**c**) Reconstruction of Sox4/Sox11-ablated cells rescued by Sox11WT or Sox11 phospho-mutants at 17dpi. The 17dpi time point was chosen because adult-born neurons feature a complex dendritic morphology at this time point. (**d**–**f**) SOX11WT and phospho-mutants differentially affect dendrite morhphogenesis. (**d**) Sholl analysis of Sox4/Sox11-ablated cells rescued by Sox11WT or Sox11 phospho-mutants (Two-way ANOVA: WT vs NON p = 7.00E-05, WT vs MIMIC p = 9.37E-38 and NON vs MIMIC p = 3.78E-10, n = 4 animals). Analysis of (**e**) dendritic length (Mann-Whitney Test: WT vs NON p = 0.83, WT vs MIMIC p = 0.03, NON vs MIMIC p = 0.03) (**f**) the dendritic branch points of Sox11WT (Mann-Whitney Test: WT vs NON p = 0.48, WT vs MIMIC p = 0.03, NON vs MIMIC p = 0.14) and (**g**) dendritic termini (Mann-Whitney Test: WT vs NON p = 0.34, WT vs MIMIC p = 0.06, NON vs MIMIC p = 0.65). (**h**) Luciferase assays using a SOX11-responsive minimal promoter n = 3, performed in HEK293T cells (Student’s unpaired t-test: WT vs NON p = 0.013, WT vs MIM p = 0.016, NON vs MIMIC p = 0.07). (**i**) Luciferase assays using Stathmin1 (Stmn1) promoter performed in HEK293T cells. (**j**) Luciferase assays using Doublecortin (DCX) promoter performed in HEK293T cells (Student’s unpaired t-test: WT vs NON p = 0.0032, WT vs MIM p = 0.0003, NON vs MIMIC p = 0.00034). Data are presented as mean ± SEM.

Next, we sought to determine whether S133 phosphorylation impacts on SOX11 function *in vivo*. SOXC protein function is required for neuronal differentiation of neural precursor cells (NPCs) in the adult neurogenic niches^1,18^. In accordance with the differentiative function of SOXC proteins and in line with previous observations^1^, conditional ablation of Sox4 and Sox11 from adult hippocampal NPCs via retroviral stereotactic injections impaired the generation of cells with a prototypic polarized dentate granule neuron morphology (CTRL 87.0 ± 1.7% vs. KO 35.8 ± 4.0%, Student’s unpaired t-test: p = 0.0005) and resulted in the generation of non-polarized cells bearing a star-like morphology with multiple short processes at 12 days post injection (dpi) (Fig. 4a and b).

We next compared the ability of wildtype SOX11 and S133 phospho-mutant SOX11, i.e., Sox11S133NON and Sox11S133MIMIC, to rescue the differentiation-deficit of Sox4/Sox11-ablated NPCs. To this end, we stereotactically injected Sox4^loxP/loxP^Sox11^loxP/loxP^ double conditional knockout mice (Sox4/11 cKO mice) with an MML-retrovirus bi-cistronically encoding for Cre-recombinase and mitoDsRed together with a MML-retrovirus bi-cistronically encoding for wildtype or mutant SOX11 and GFP. Animals were sacrificed 17 days post injection to test the rescue capacity of the mutants and to validate the dendritic morphogenesis of the transduced cells. Expression of mitoDsRed and GFP was used to identify Cre-recombinase and SOX11 transduced cells, respectively. Virtually all Cre-recombinase expressing neurons co-transduced with Sox11WT, Sox11S133NON, or Sox11S133MIMIC, expressed the neuronal marker and SOX11 target gene doublecortin (>98% in all groups). Moreover, double-transduced cells featured the typical polarized dentate granule (DG) neuron morphology with an apical dendrite spanning the granule cell layer and initiation of dendrite branching in the molecular cell layer irrespective of whether they were transduced with wildtype or phospho-mutant SOX11. These data indicate that wildtype and phospho-mutant SOX11 are functional and can rescue the SoxC-loss-of-function induced neuronal differentiation deficits (Fig. 4c). A more detailed analysis, however, revealed differences in dendrite morphologies between cells rescued by wildtype or phospho-mutant SOX11 (Fig. 4c–g). Sholl-analysis showed that dendrite complexity was consistently lowest in Sox11S133MIMIC-expressing, intermediate in Sox11S133NON-expressing, and highest in Sox11WT-expressing DG neurons (Fig. 4d). The total dendritic length and number of branch points were comparable between Sox11WT- and Sox11S133NON-expressing neurons but were significantly lower in Sox11S133MIMIC-expressing neurons (Fig. 4e and f). Moreover, Sox11S133MIMIC-expressing neurons showed a strong trend towards lower numbers of dendritic termini (Fig. 4g). The observation that SOX11 wildtype and S133 phospho-mutants elicited striking differences in dendrite morphology suggests that S133 phosphorylation modulates SOX11 activity/function.

To begin to understand the mechanistic basis of the differential effects of the S133 phospho-mutants on dendrite morphology, we compared their ability to transactivate different target genes. Specifically, we compared the ability of the mutants to activate the SOX11-responsive minimal promoter and the promoters of Stathmin1 (Stmn1) and Doublecortin (DCX). Both SOX11S133 phospho-mutants showed increased transactivation of the SOX11-responsive minimal promoter compared to the SOX11WT (Fig. 4h). In addition, while Sox11WT, Sox11S133NON, and Sox11S133MIMIC, showed comparable abilities for activation of the Stmn1 promoter^19,20^ (Fig. 4i), both phospho-mutants consistently activated the DCX-promoter^1^ more strongly than SOX11WT with the phospho-mimetic mutant being the most potent transactivating form (Fig. 4j). These results raise the interesting possibility that the phosphorylation of SOX11 on S133 may modulate the transactivation activity of SOX11 on a subset of target genes.

## Discussion

SOX11 has been firmly established as a critical developmental transcriptional regulator with pleiotropic functions in early and late processes of neurogenesis^21^. The regulation of SOX11, however, remains poorly understood. Extending our previous work that identified ten phosphorylatable serine residues in the SOX11 protein^11^, we show that the three N-terminal serines are important for SOX11’s transcriptional activity. We also demonstrate that one of them, S133, is phosphorylated by the cyclic AMP responsive PKA kinase. Most importantly, *in vivo* replacement experiments in developing adult-generated hippocampal neurons revealed that the phospho-mimetic and non-phosphorylatable S133 mutants differentially affect neuronal morphogenesis, indicating that phosphorylation on S133 modulates SOX11’s activity or function.

Mutation analysis of the predicted phospho-serines suggested that the three N-terminal phospho-serines are important for SOX11’s transcriptional activity. Interestingly, while the combined mutation of all three N-terminal serines into phospho-mimetic or non-phosphorylatable amino acids abolished transcriptional activity, single mutants of S30 (phospho-mimetic) and S133 (phospho-mimetic and non-phosphorylatable) can activate transcription. This could be potentially explained by S137, which may further contribute to the ability of SOX11 to transactivate. It is also possible that a distinct combination of phosphorylations on the three N-terminal serines may be required for transcriptional activity. Given that the N-terminal serines surround the DNA-binding HMG box, it is also tempting to speculate that the combinatorial phosphorylation of these serines determines the affinity of SOX11 to DNA. The function of the seven C-terminal phosphorylatable serine residues remains to be established. Mutation of all seven C-terminal serines to a phospho-mimetic or a non-phosphorylatable form did not affect the transcriptional activity of SOX11 in luciferase reporter assays. These basic transcriptional assays, however, do not exclude a functional relevance for the phosphorylation status of the C-terminal serine residues in the regulation of SOX11’s transcriptional activity. Further studies are required to determine whether, e.g., a combinatorial phosphorylation code involving the C-terminal serine residues, may fine-tune transcriptional activity, modulate target gene selection, or influence interaction with other transcription factors.

We explored the impact of the phosphorylation status of SOX11 on S133 in the context of adult hippocampal neurogenesis. In the adult hippocampal dentate gyrus a tightly regulated transcription factor sequence that includes SOX11, generates new functional dentate granule neurons^22–24^. Adult-generated hippocampal DG neurons feature a highly stereotypic dendritic morphology, which is essential for DG neuron function and whose perturbation is associated with deficits in hippocampus-dependent information processing and behavior^25–29^. Having confirmed that SOXC transcription factor function is essential for neuronal differentiation in the adult neurogenic lineage^1^, we found that SOX11 and its S133 phospho-mutants were equally potent in rescuing the neuronal differentiation deficit of SoxC-deficient cells *in vivo*. Intriguingly, in-depth analysis of rescued cells revealed that the SOX11 WT and S133 phospho-mutants differentially impacted on dendrite morphogenesis. These observations raise the intriguing possibility that S133 phosphorylation may function to fine-tune dendrite development. The molecular mechanism through which S133 phosphorylation modulates dendrite development remains to be established. Interestingly, SOX11WT and the SOX11S133 mutants differentially activated select promoters of genes encoding for cytoskeleton-modifying proteins, suggesting that S133 phosphorylation may allow to modulate the expression of a defined subset of genes involved in the regulation of dendrite development. The finding that expression of either SOX11 S133 phospho-mutant produced a dendritic phenotype that was different from the SOX11WT phenotype raises the intriguing possibility, that development of the stereotypic dentate granule cell morphology requires a precise balance between the phosphorylated and un-phosphorylated S133 form of SOX11.

We provide evidence that the S133 residue is targeted by PKA. cAMP/PKA-signaling is a central signaling pathway in neuronal development, which in adult neurogenesis is involved in the control of neuronal fate determination, neuronal survival, and neuronal morphogenesis^30–33^. cAMP response element binding protein (CREB) is considered the primary transcriptional mediator downstream of cAMP/PKA-signaling regulated neuronal development. Our findings raise the intriguing hypothesis that at least some aspects of PKA-regulated neuronal development such as dendritic development, are mediated through a transcription factor network that includes CREB and SOX11.

There is evidence that impaired generation and functional integration of adult-born DG neurons as well as dys-regulation of cAMP/PKA-signaling play a significant role in the pathophysiology of neuropsychiatric and neurodegenerative disorders such as anxiety, depression, and Alzheimer’s disease^34–38^. It will be interesting to explore if and how dys-regulation of SOX11 phosphorylation contributes to those pathologies.

## Material and Methods

### Animal experiments

Animal experiments were conducted in accordance with the European Communities Council Directive (86/609/EEC). Animal experiments were approved by the review boards of the governments of Upper Bavaria (Regierung von Oberbayern) and Middle-Franconia (Regierung von Mittelfranken). All mice were group-housed under a 12 h light/dark cycle with *ad libidum* access to food and water. C57Bl/6NRj mice, were obtained from Janvier Labs (Le Genest-Saint-Isle, France). Mice of mixed 129SvJ/C57Bl6J background homozygous for conditional alleles for Sox4^loxP^^39^ and Sox11^loxP^^40^ were used to perform the loss-of-function experiments. Male and female littermates were included in the analysis.

### Retrovirus preparation

Mouse moloney leukemia (MML) retroviruses were used to specifically transduce proliferating cells, i.e., neural precursor cells (NPCs) in the adult hippocampal dentate gyrus^41^. The pCAG–GFP and pCAG–mitoDsRed retroviral plasmids have been described previously^24,42^. For retrovirus-mediated expression of wildtype and S133-phospho-mutant Sox11, the mouse cDNA was cloned into the pCAG–IRES–GFP^30^ to generate pCAG–Sox11WT–IRES–GFP^1^, pCAG–Sox11S133NON–IRES–GFP and pCAG–Sox11S133MIMIC–IRES–GFP, respectively. The pCAG–GFP–IRES–Cre was generated from the pCAG–GFP vector^24^ by replacing the GFP coding sequence with cDNA for GFP and Cre recombinase as well as an internal ribosomal entry site (IRES). The pCAG–Cre–IRES–mitoDsRed was generated from the pCAG–mitoDsRed vector^30,42^. Retroviruses were generated as described previously^43^. Viral titers used ranged between 0.1 to 2 × 10^7^ colony-forming units (CFU)/ml.

### Stereotactic injections

Seven-week-old mice of mixed 129SvJ/C57Bl6J background carrying the conditional alleles for Sox4^loxP^^39^ and Sox11^loxP^^40^ were housed with running wheels for a week to enhance proliferation of NPCs^41^. Subsequently, mice were deeply anesthetized with a mixture of ketamine (100 mg/kg bodyweight) and xylazine (10 mg/kg bodyweight). Mice were stereotactically injected with 1 μl of 1:1 retroviral mixtures (CAG-GFP-IRES-CRE + CAG-mitoDsRed; CAG-CRE-IRES-mitoDsRed + CAG-Sox11WT-IRES-GFP; CAG-CRE-IRES-mitoDsRed + CAG-Sox11S133NON-IRES-GFP; CAG-CRE-IRES-mitoDsRed + CAG-Sox11S133MIMIC-IRES-GFP) into the left and right dentate gyrus (coordinates: from bregma −1.9 mm anterior/posterior, +/−1.6 mm medial/lateral, −1.9 mm dorsal/ventral from dura).

### Tissue preparation, Immunofluorescence stainings, Imaging

For histological analyses of adult mice, animals were killed with CO_2_. Subsequently, animals were perfused transcardially with phosphate-buffered saline (PBS, pH = 7.4) followed by 4% paraformaldehyde (PFA, in Phosphate buffer, pH = 7.4). Brain tissue was removed, post-fixed in 4% PFA overnight, and dehydrated in 30% sucrose solution prior to slicing (50 μm and 100 μm thick) at a sliding microtome (Leica Microsystems, Wetzlar, Germany). For immunofluorescence stainings free-floating sections were used. Sections were washed six times with Tris-buffered saline (TBS; 25 mM Tris/HCl, 137 mM NaCl, 2.6 mM KCl) incubated in blocking solution (TBS, 10% normal donkey serum, 0.25% TritonX100) for one hour. After 72 h incubation with primary antibodies (Table 1) diluted in blocking solution, sections were washed six times in TBS and incubated with fluorophore-coupled secondary antibodies (Table 2) overnight. Sections were incubated in DAPI (5 ng/ml in PBS) for nuclear staining, washed three times in TBS, and mounted in Aqua Polymount (Polysciences, Warrington, PA, USA) for imaging.

**Table 1:** Primary antibodies.

Antigen	Species	Company	Working Dilution	Catalog number	RRID
SOX11	Rabbit	Abcam	1:500	ab134107	AB_2721126
Doublecortin (DCX)	Goat	Santa Cruz	1:500	SC8066	AB_2088494
Mouse SOX11*	Rat	In house	1:100	—	AB_2732801
GFP	Chicken	Aves Labs	1:500	GFP-1020	AB_10000240
pCREB(S133)	Rabbit	Cell Signaling	1:500	9198 S	AB_2561044
PKA Catalytic Subunit	Rabbit	Abcam	1:500	ab75991	AB_1524202
PKA Catalytic Subunit	Mouse	Santa Cruz	1:500	sc28315	AB_628136
RFP	Rat	Chromotek	1:500	5F8-100	AB_2336064

*The monoclonal Sox11 antibody (clone 19B2, IgG2a, AB_2732801) was generated in house (E. Kremmer, Service Unit Monoclonal Antibodies, Helmholtz-Zentrum München) as previously described^51^. Briefly, as antigen, HIS-GST fusion protein using the pETM30 vector (provided by the EMBL) was generated encompassing the homologous mouse sequence of a human SOX11 PrEST (https://www.proteinatlas.org)^52^ and corresponding to the amino acids 189-301 of mouse SOX11. The protein was recombinantly expressed in E. coli. The soluble protein fraction was purified via the N-terminal 6xHis tag using Ni-NTA Agarose (Qiagen) according to standard protocols provided by the manufacturer and followed by dialysis against PBS to remove the imidazole. Rats (Lou/C) were immunized with purified His-GST Sox11-189-301. Fusion was performed with the myeloma cell line P3X63Ag8.653. After generation of hybridomas, selected clones were subcloned till stability.

For histological analyses of embryonic mouse brains, timed pregnant mice were killed by cervical dislocation. E15.5 embryos were removed, and their heads were fixed in 4% PFA overnight and then transferred to 30% sucrose in PBS overnight for dehydration. Embryonic tissues were embedded in freezing media (Jung, Nussloch) and cut in 10 µm thin sections with a cryotome (Leica Microsystems, Wetzlar). The sections were transferred on slides, dried for 2 h at room temperature and stored at −80 °C. For immunofluorescence stainings sections were washed with 1x PBS, permeabilized in 0.1% Triton-X/PBS and blocked with blocking solution (10% FCS, 1% BSA in PBS) at room temperature for 2 h in a wet chamber. Subsequently, tissue was incubated overnight with primary antibodies (Table 1) diluted in blocking solution at 4 °C. Slides were washed several times with 1x PBS and incubated with secondary antibodies (Table 2) diluted in blocking solution for 2 h at room temperature. The sections were washed three times with 1x PBS. The nuclei were stained with (5 ng/ml in PBS) DAPI (4′,6-diamidino-2-phenylindole) for 2 minutes. The sections were then washed with 1x PBS for 10 minutes. The slides were mounted with 50 µl Mowiol (Sigma-Aldrich) and stored at 4 °C. Imaging for both adult and embryonic slices was conducted with a Zeiss LSM 780 equipped with four lasers (405, 488, 559, and 633 nm) and 40x and 63x objectives. The confocal single plane and z-projection images were processed using the Fiji ImageJ software^44^.

### Antibodies

The following antibodies and conditions were used for this

**Table 2:** Secondary antibodies.

Antibody	Host	Company	Working Dilution	Catalog number	RRID
Alexa488-coupled anti rabbit IgG	Donkey	Life Technologies	1:1,000	A21206	AB_141708
Cy3-coupled anti rat IgG	Donkey	Jackson	1:1,000	712-165-153	AB_2340667
Cy5-coupled anti goat IgG	Donkey	Jackson	1:1,000	705-175-147	AB_2340415
Alexa488-coupled anti chicken IgG	Donkey	Biotium	1:1,000	20166	AB_10854387
Horseradish Peroxidase (HRP)-coupled anti rat IgG	Goat	Jackson	1:1,000	112-035-003	AB_2338128
Horseradish Peroxidase (HRP)-coupled anti mouse IgG	Goat	Jackson	1:1,000	115-035-003	AB_10015289

study:

### Morphological Analysis

Coronal brain slices from the stereotactically injected mice were stained with anti-GFP and anti-RFP antibodies and imaged with a confocal microscope Zeiss LSM 780 equipped with four lasers (405, 488, 559 and 633 nm) and 40x and 63x objectives. Double-positive cells were reconstructed with Imaris Software (Bitplane). In total, a minimum of 18 cells from 4 animals were analyzed per group.

### Plasmids

Mouse Sox11 wildtype and mutants were cloned into the pEGFP-C3 (Clontech) as previously described^11^. Sox11 mutants (C3-Sox11p^MIMIC^, C3-Sox11p^NON^, Sox11S133NON, and Sox11S133MIMIC) were synthesized by Thermo Fisher Scientific GENEART GmbH, Germany and cloned into the pEGFP-C3 plasmid. All other mutants were cloned by restriction enzyme digestions of the original mutants and the wt Sox11. For phosphosite mapping, Sox11 was subcloned into the pDEST-NSF vector^45^ to produce an N-terminally Strep-Flag tagged SOX11^11^. The plasmid with the minimal Sox11-responsive promoter containing 3xSox11 binding sites in front of the luciferase gene was previously described^12^. DCX reporter construct (Doublecortin promoter was cloned in front of the luciferase gene in the PGL3 construct -Promega-)^46^, Stmn1 reporter construct (Stathmin1promoter was cloned in front of the luciferase gene in the PGL3 construct -Promega-)^19,20^ and Renilla–construct under the control of the human elongation factor 1 promoter^47^.

### Tissue culture, transfection, immunofluorescence stainings, and reporter assays (Luciferase assays)

For transfection, HEK293T cells (ATCC, Wesel, Germany; CRL-3216) were seeded in 24-well plates at a density of 6 × 10^4^ cells per well. 24 h later, cells were transfected with jetPEI (Polyplus transfection, 101-10 N) according to the manufacturer’s protocol with equal amounts (0.05 μg/well) of the expression vectors (C3, C3-Sox11p^WT^, C3-Sox11p^NON^, C3-Sox11p^MIMIC^, C3-Sox11p^N3W7^, C3-Sox11p^M3W7^, C3-Sox11p^W3N7^, C3-Sox11p^W3M7^) together with Sox11-responsive minimal promoter Luciferase reporter construct (0.05 μg/well) and a Renilla–luciferase under the control of the human elongation factor 1 promoter (0.005 μg/well)^47^. Alternatively, the cells were transfected with 0.05 μg/well of pCAG–GFP or pCAG-Sox11WT-IRES-GFP or pCAG-Sox11S133NON-IRES-GFP or pCAG-Sox11S133MIMIC-IRES-GFP together with either DCX–promoter Luciferase reporter construct (0.05 μg/well)^46^ or with Stathmin1- promoter Luciferase reporter construct (0.05 μg/well)^19,20^ and a Renilla–luciferase under the control of the human elongation factor 1 promoter (0.005 μg/well)^47^. Cell were analyzed 48 h after transfection using the Promega dual luciferase kit and a Centro LB 960 luminometer. Luciferase assays were performed from at least three biological replicates.

For immunofluorescence stainings, HEK293T cells were seeded in 24-well plates with coverslips at a density of 60,000 cells per well. The cells were transfected with jetPEI (Polyplus transfection, 101-10 N) according to the manufacturer’s protocol with 0.5 μg/well of each of the following plasmids: pCAG–Sox11WT–IRES–GFP^1^, pCAG–Sox11S133NON–IRES–GFP and pCAG–Sox11S133MIMIC–IRES–GFP, respectively. 48 h after transfection the cells were fixed with 4% PFA, washed with 1x PBS and incubated for one hour in blocking solution followed by an overnight incubation with primary antibodies (Table 1) in blocking solution. Subsequently, the cells were washed with blocking solution and incubated in the dark, at room temperature for two hours with secondary antibodies (Table 2) diluted in blocking solution. The cells were incubated with DAPI (to indicate the nucleus) for 10 min and then washed repeatedly with PBS. Finally, the coverslips were mounted in Aqua Polymount (Polysciences, Warrington, PA, USA) for imaging. A Zeiss LSM 780 confocal microscope equipped with four lasers (405, 488, 559 and 633 nm) and 40x and 63x objectives were used to take single plane and z-projection images. Image processing was done in Fiji ImageJ^44^. The nuclear or nuclear and cytoplasmic localization of SOX11 was determined with the use of the nuclear marker DAPI by line intensity plots.

### Co-immunoprecipitation assay and Western Blot

Timed pregnant mice were killed by cervical dislocation and the brains from E15.5 embryos were immediately processed. Brains were lysed in Lysis buffer [10 mM HEPES pH 7.9, 10 mM KCl, 0.2Mm EDTA, protease inhibitor EDTA free cocktail (Roche PVT GmbH Waiblingen, Germany) and Phosphatase Inhibitors Cocktail (Sigma Aldrich Chemie GmbH Munich, Germany)] with the use of a tissue homogenizer. 1% Nonidet P-40 was added and samples were vortexed. Following the addition of 150 mM NaCl, samples were subjected to 15 min of rotating incubation at 4 °C. An appropriate amount of the lysate was kept as input. The remaining lysate was split and incubated with the respective antibodies. After an overnight incubation 25 μl of 1:1 Protein G Sepharose 4 Fast Flow, 17-0618-01 (GE Healthcare Bio-Sciences AB, Uppsala, Sweden): Lysis Buffer was added. After 4 h of incubation at 4 °C on a rotating shaker, samples were washed at least 5 times by centrifugation at 1,600rcf, removal of the supernatant and addition of fresh lysis buffer. Coupled beads were incubated with 3x Laemmli Buffer for 5 minutes at 95 ^o^C. Proteins were separated in a 10% SDS-PAGE gel. Gels underwent wet transfer onto nitrocellulose membrane. Membranes were blocked in 5% ^w^/_v_ skim milk (Sigma Aldrich) in TBS with 0.1% Tween 20 (TBS-T). Incubation with primary antibodies diluted in blocking solution was performed overnight at 4 °C, and was followed by washing with TBS-T. Secondary antibodies were diluted in blocking solution and incubated with the membranes for at least 1 h at room temperature followed by washing with TBS-T. The membranes were visualized by Clarity Western enhanced chemiluminescence (ECL) Substrate (Bio-Rad) with ChemiDoc XRS + System (Bio-Rad). Images were processed via ImageLab 5.2.1 Setup (Bio-Rad).

### ***In vitro*** phosphorylation assay

HEK293T cells were seeded in ten 10 cm dishes at a density of 8 × 10^5^ cells per plate. The following day the cells were transfected with jetPEI (Polyplus transfection, 101-10 N) according to the manufacturer’s protocol with 3 μg/plate of pDEST-NSF-Sox11. 48 h after transfection the cells were washed twice with 1x PBS, scraped from each plate using 1 ml PBS and collected together for lysis. The cells were lysed in Lysis Buffer and incubated with anti-SOX11 Rb -previously validated^11^- antibody overnight. Extracts were incubated with Sepharose Beads for 4 h followed by multiple washing steps (as described in the Co-immunoprecipitation assay section). The beads were finally washed four times with Kinase Buffer [25 mM Tris (pH 7.5), 5 mM β-glycerophosphate, 2 mM DTT, 0.1 mM Na_3_VO_4_, 10 mM MgCl_2,_ protease inhibitor EDTAfree cocktail (Roche) and Phosphatase inhibitors cocktail (Sigma Aldrich)] and resuspended in 80 μl of Kinase Buffer supplied with 400 μM ATP. In one third of the immunoprecipitated SOX11 100 nM of purified human PKAc_alpha_ (a kind gift from Friedrich Herberg, University of Kassel, Germany) was added and in the other third we added 100 nM PKAc_alpha_ together with 50 μM PKI (a kind gift from Friedrich Herberg). The samples were incubated at 37 ^o^C for 60 min. Finally, the samples were snap frozen and further analyzed by Mass Spectrometry.

### Interactomic analysis (AP-MS)

For the identification of SOX11 interaction partners, Strep-FLAG-tagged mouse SOX11 was transiently expressed in SILAC-labelled (light: Lys-0/Arg-0; heavy: Lys-8/Arg-10) Neuro2a cells. The isolation of SOX11 protein complexes was performed by co-IP from nuclear extracts. Confluent cells were washed with PBS (Life Technologies), harvested, washed again and pelleted. Subsequently, the cytoplasmic fraction was collected using 3 times the volume of buffer A [10 mM HEPES, 1 mM EDTA, 0.1 mM EGTA, 10 mM KCl, 1 mM PMSF, 1 µg/ml phosphatase inhibitor cocktail 2 and 3 (Sigma Aldrich), 1 mM DTT, 1 µg/ml complete protease inhibitor (Roche)] according to pellet size. After 15 min of incubation 0.1% NP40 (Roche) was added to break the cell membrane. Following centrifugation, the nuclei were harshly resuspended in 2 times volume according to pellet size buffer C [20 mM HEPES, 0.2 mM EDTA, 0.1 mM EGTA, 25% glycerol, 420 mM NaCl, 1.5 mM MgCl_2_, 1 mM PMSF, 1 µg/ml phosphatase inhibitor cocktail 2 and 3 (Sigma Aldrich), 1 mM DTT, 1 µg/ml complete protease inhibitor (Roche)] and rotated for 20 min at 4 °C. After centrifugation, the supernatant containing nuclear proteins was collected. Flag affinity purification was performed applying anti-Flag M2 agarose beads (Sigma). Beads were washed 3 times in buffer C, thereof twice without phosphatase and protease inhibitors, thereafter 50 µl packed beads were incubated 1.5 h at 4 °C on an end-over-end shaker with 3.5 to 6 mg nuclear extract, supplemented with 300U/mg Benzonase Nuclease (Novagen) to exclude DNA-mediated interactions. The bead/lysate mixture was washed 3 times with wash buffer [TBS buffer (30 mM Tris-HCl, pH 7.4, 150 mM NaCl) containing 0.1% NP40, 1 µg/ml phosphatase inhibitor cocktail 2 and 3 (Sigma Aldrich), 1 µg/ml complete protease inhibitor (Roche)] avoiding dehydration of the matrix. Finally, bait proteins were eluted through affinity-based exchange by incubation with 200 µl FLAG elution buffer [200 µg/µl FLAG peptide (Sigma Aldrich) in TBS buffer] for 10 min under shaking at 4 °C. Corresponding heavy and light-labelled eluates were combined subsequently. Mass spectrometry was performed on an LTQ Orbitrap Velos instrument (Thermo Scientific) as described previously^48^. MS raw data were analyzed with MaxQuant V.1.5.0.30^49^ using the SwissProt mouse database (2014_04, 16,669 entries) as previously described^50^. Carbamylation was set as fixed modification, Glutamine/Asparagine deamination, methionine oxidation and protein N-terminal acetylation were included as variable modifications. Min ratio count was set to one. Only protein IDs found in a minimum of one forward and one reverse experiment with a minimum of one unique peptide have been considered for the final dataset. Significance A and B were calculated according to Cox and Mann^49^ by application of in-house R-scripts. For the analysis, three forward and three reverse (label switch) experiments were considered (total N = 6).

### Line Intensity Plots

Comparative line intensity plots of the fluorescence of DAPI, a nuclear label, and of SOX11 immunofluorescence, were used to demonstrate SOX11’s subcellular localization. The Plot Profile function of Image J^44^ on a single plane z-stack of confocal microscopy pictures was used to produce the data plotted on the line intensity plots.

### Statistics

Mann-Whitney-Test (GraphPad Prism version 7.00 for Windows, GraphPad Software, La Jolla California USA, www.graphpad.com), unpaired Student’s T-test, or Two-Way-Anova (Microsoft Excel) were applied to assess statistical significance of the differences between the experimental groups. For the morphological analysis of the rescued cells at least 4 cells from 4 animals per experimental group were analyzed. The average value for each animal was calculated and used to analyze differences between experimental groups.

### Prediction of protein kinase A phosphorylation sites

SOX11 Mus Musculus protein sequence was analyzed with pkaPS-Prediction of protein kinase A phosphorylation sites using the simplified kinase binding model, http://mendel.imp.ac.at/pkaPS/ ^17^.

## Electronic supplementary material

Supplemental Figure 1

Supplemental Table 1
