# Supplementary material for: Phosphorylation of the neurogenic transcription factor SOX11 on serine 133 modulates neuronal morphogenesis
Source: Sci Rep. 2018 Nov 1;8:16196. doi: 10.1038/s41598-018-34480-x (PMC6212486; doi:10.1038/s41598-018-34480-x)
Supplement: Supplementary file 1 — Supplemental Figure 1 [file 41598_2018_34480_MOESM1_ESM.pdf]

Supplemental Figure 1

Phosphorylation of the neurogenic transcription factor SOX11 on serine 133 modulates neuronal morphogenesis

Elli-Anna Balta[1], Iris Schäffner[1], Marie-Theres Wittmann[1], Elisabeth Sock[1], Felix von Zweydorf[2], Julia von Wittgenstein[1], Kathrin Steib[3], Birgit Heim[4], Elisabeth Kremmer[5], Benjamin Martin Häberle[1], Marius Ueffing[4], Dieter Chichung Lie[1,6*], Christian Johannes Gloeckner[2,4,6*]

[1] Institute of Biochemistry, Friedrich-Alexander Universität Erlangen-Nürnberg, 91054 Erlangen, Germany
[2] DZNE-German Center for Neurodegenerative Diseases, 72076 Tübingen, Germany
[3] Institute of Developmental Genetics, Helmholtz Zentrum München, German Research Center for Environmental Health, 85764 Neuherberg, Germany
[4] University of Tübingen, Institute for Ophthalmic Research, Center for Ophthalmology, 72076 Tübingen, Germany
[5] Monoclonal Antibody Core Facility, Helmholtz Zentrum München, German Research Center for Environmental Health, 85764 Neuherberg, Germany
[6] equal contribution

Supplemental Figure 1

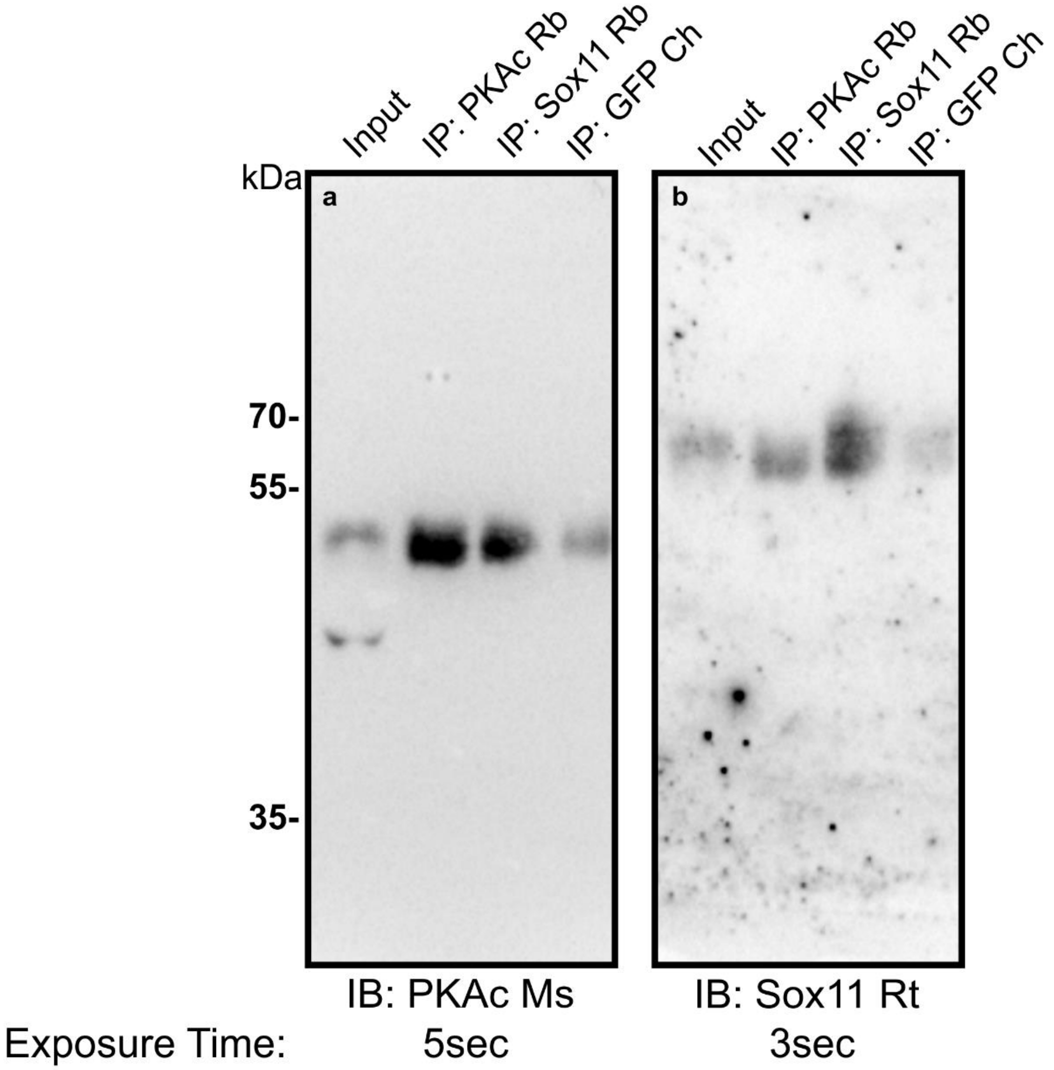

Supplemental Figure 1: Full blots from Figure 2d.
The membrane was blotted with an anti-PKA mouse (Ms) antibody. The picture was taken 5 seconds after exposure (a). The same membrane was sequentially blotted with an anti-SOX11 rat (Rt) antibody. The picture was taken 3 seconds after exposure (b).
